# Advances in Spatial Transcriptomics in Bone

**DOI:** 10.1007/s11914-025-00949-8

**Published:** 2026-01-15

**Authors:** Nico Valerio Giger, Esther Wehrle

**Affiliations:** 1 AO Research Institute Davos, Clavadelerstrasse 8, 7270 Davos Platz, Switzerland; 2 Department of Health Sciences and Technology, ETH Zurich, Zurich, 8092 Switzerland

**Keywords:** Spatial transcriptomics, Bone, Fracture healing, Bone development, Multi-omics, Sample preparation

## Abstract

**Purpose of Review:**

Spatial transcriptomics enables to capture the whole transcriptome within the local microenvironment in bone. Within this review, we provide an overview of recent spatial transcriptomics applications and indicate its potential for advancing basic and translational research in skeletal development and maintenance, bone-related disorders, as well as fracture healing.

**Recent Findings:**

Recent developments in sample preparation protocols have enabled the application of spatial transcriptomics to bone. However, there is a lack of standardized data analyses pipelines for spatial transcriptomics in bone. The interpretability of current data sets further suffers from low sample sizes.

**Summary:**

We consider spatial transcriptomics a cornerstone technology for unravelling bone’s spatial and molecular complexity. Via integration into emerging multi-omics and multi-modal imaging approaches, spatial transcriptomics has the potential to advance translational-targeted research in the fields of orthopaedics and musculoskeletal research.

## Introduction

RNA sequencing technologies have advanced our understanding of transcriptional regulation in bone [1]. While bulk approaches deliver the transcriptome of homogenized tissue, single-cell RNA sequencing (scRNA-seq) technologies take into account the heterogeneity of cells, enabling the investigation of cell-fate transitions as well as cell‒cell interactions within the tissue [2, 3]. However, despite its unprecedented resolution, scRNA-seq inherently lacks spatial context. Traditional histological methods preserve morphology [4] and spatial information but lack high-dimensional molecular profiling [5–7]. Emerging spatial transcriptomics (ST) technologies integrate sequencing and imaging approaches, allowing to simultaneously capture the whole transcriptome and spatial localization within intact tissue architecture [8]. Specifically, ST enables spatially resolved mapping of cellular gene expression within the local microenvironment, offering insight into tissue organization, functional heterogeneity and cellular interactions that are inaccessible to dissociative methods [9–13]. ST was selected as ‘Method of the Year 2020’ by Nature Methods [5], further highlighting its potential to advance research.

ST is rapidly evolving, driven by advances in sample preparation protocols [14–16], technology platforms [10, 13] as well as computational tools [6, 17–28]. Benchmarking studies have been instrumental in delineating strengths and limitations of specific ST approaches, guiding researchers in selecting adequate strategies for specific biological questions [29–32].

ST has been successfully applied to a variety of soft tissues driving the development of tissue- and organ-specific spatial atlases for mouse and human settings [33, 34]. Application of ST to bone has been challenging due to bone’s dense, calcified matrix impacting preservation of tissue morphology and RNA quality during sample preparation [35]. First ST applications within orthopaedics and musculoskeletal research have therefore been described for non- and lowly mineralized tissue such as muscle, joint, tendon and synovium derived from preclinical and clinical samples (for review see: [36, 37]). Recent optimizations in sample pre-treatment and decalcification protocols [14–16] have extended the application of ST to fully mineralized samples. These advances enabled the study of bone’s molecular features under both healthy and impaired conditions (see Table 1).

**Table 1:** Applications and advances of ST in bone

Category	Species	Sample	Fixation & decal.	Embedding*	ST platform	Study design & groups	Sample size	Main study outcome and advances	Reference
Bone development and maintenance	Mouse	Femur	PFA	Cryo	LCM-seq	Sinusoids vs. endosteum vs. no vessels vs. sub endosteal	ST: *n* = 11–28 per region	By combining scRNAseq and ST, the molecular, cellular and spatial bone marrow niche organization was revealed	Baccin et al. 2020 [42]
	Mouse	Calvaria	-	OCT (Cryo)	Visium	TrkA^F592A^ vs. controls	Total: *n* = 8/group ST: *n* = 5–7/group	ST revealed linkage of sensory nerves to BMP/TGF-β signaling in cranial suture patency	Tower et al. 2021 [46]
	Mouse	Head	Formalin	Paraffin	Visium	Embryos, E14.5 vs. E15.5	Total: *n* = 3/group ST: *n* = 3/group	By combining bulk/snRNAseq and ST, a shift in transcriptional programming was linked to osteogenesis of the secondary palate	Piña et al. 2023 [47]
	Mouse	Femur	PFA, EDTA	Paraffin	GeoMx	Bone marrow, Young vs. old	Total: *n* = 1/group ST: *n* = 1/group	ST enabled sinlge cell resolution indicating a possible effect of spatial location on megakaryocyte heterogeneity	Tilburg et al. 2023 [43]
	Mouse	Femur	Formalin, EDTA	Paraffin	Visium	Femur, compartment analyses	ST: 2 sections from 2 mice	By combing ST with published scRNAseq data, niche analyses of skeletal stem & progenitor cell subtypes were performed	Xiao et al. 2023 [44]
	Mouse	Hindlimb	Formalin, Formic acid	Paraffin	Visium	Growth plate, Col2Cre; Adgrg6f/f vs. wildtype P20	Total: *n* = 3/group ST: *n* = 1/group	ST revealed the G protein-coupled receptor ADGRG6 to be essential for maintaining chondrocyte homeostasis	Bian et al. 2024 [48]
	Human	Limb	-	OCT (Cryo)	Visium	Human embryonic limb PCW5.6–8.1	ST: *n* = 1–4/group	By combining scRNAseq and ST, limb development, cell diversification & cross-species conservation were demonstrated	● Zhang et al. 2024 [49]
	Human	Spine	-	OCT (Cryo)	Visium, CARTANA HS	Embryonic cervical, thoracic & lumbar spine, PCW7-9	ST: *n* = 2/group	By combining scRNAseq and ST, a developmental spine atlas was created shedding light on *HOX* gene expression	Lawrence et al. 2024 [51]
	Human	Organoid	-	OCT (Cryo)	Tomo-seq	Organoids (WTS2 cell line) d7	Total: *n* = 3 ST: *n* = 2	Via scRNAseq and ST, a self-organizing model of trunk organo-genesis was shown to recapitulate cord/spine co-morphogenesis	Gribaudo et al. 2024 [52]
	Human	Limb and cranium	-	OCT (Cryo)	Visium CytAssist, CARTANA HS	Embryonic limb & cranium PCW 5–11	Total: *n* = 12 ST: *n* = 1–3/group	Via combining sequencing and imaging based technologies, a multi-omic atlas of embryonic development was created	● To et al. 2024 [50]
Bone disorders	Human	Bone tumor	Formalin	Paraffin	GeoMx DSP	Bone metastasis, lytic and blastic prostate cancer	ST: *n* = 3/group	ST revealed distinct tumor microenvironments of lytic and blastic bone metastases in prostate cancer patients	Ihle et al. 2019 [53]
	Human	Chordoma	-	OCT (Cryo)	Visium	Primary and recurrent chordoma	Total: *n* = 6 ST: *n* = 3	By combining scRNAseq and ST, cancer associated fibroblast (CAF) subpopulations for chordoma progression were revealed	Zhang et al. 2024 [54]
	Human	Femur	Formalin, EDTA	Paraffin	Visium CytAssist	Femoral head from OA patient	Total: *n* = 1 ST: *n* = 1	By combining scRNAseq and ST, a high-resolution atlas of human bone and bone marrow was presented	Lin et al. 2025 [55]
	Human	Chordoma		OCT (Cryo)	Visium	Sacral chordoma	Total: *n* = 126 ST: *n* = 2	By combining bulk/scRNAseq and ST, the role of inflammatory CAFs in chordoma progression was unveiled	Zheng & Guo 2025 [56]
Fracture healing	Mouse	Digit	-	OCT (Cryo)	Visium	Digit amputation, young/aged/aged con/OAA-aged	ST: *n* = 4–7/group	ST revealed metabolic changes underlying age-dependent declines in digit regeneration	Tower et al. 2022 [61]
	Mouse	Femur	PFA, EDTA	Paraffin	Visium CytAssist	Femur osteotomy, MDA-MB-231 vs. vehicle	Total: *n* = 1–2/group ST: *n* = 1 2/group	ST showed impaired fracture healing in metastatic breast cancer associated with downregulation of matrix/structural regulators	Jiang et al. 2024 [64]
	Mouse	Tibia	Formalin, EDTA	Paraffin	Visium	Tibia osteotomy, *Nf1* ^Postn^ *vs. Postn *cre^−^;*Nf1*^+/fl^	Total: *n* = 2 3/group ST: *n* = 2–3/group	ST implicated impaired BMP signaling in NF1 fracture pseudarthrosis	Rios et al. 2024 [66]
	Mouse	Calvaria	-	OCT (Cryo)	Visium	Calvarial defect, Col hydrogel vs. nHA solution vs. Col + nHA	Total: *n* = 6/group ST: *n* = 3/group (Col + nHA vs. empty)	By combining scRNAseq and ST, functional MSCs with high expression of matrix Gla protein were identified, which may serve as pioneer subpopulation involved in bone repair	Wan et al. 2024 [67]
	Mouse	Femur	Formalin, EDTA	Paraffin	Visium	Femur defect, loaded vs. 0 N controls	Total: *n* = 2/group ST: *n* = 1/group	Via linking the ST derived transcriptome to L*iv*E, spatial profiles in high/low strained callus regions were characterized	● Mathavan et al. 2025 [70]
	Mouse	Mandibula	PFA, EDTA	Paraffin	GeoMx	Mandibular fracture, hypochondroplasia vs. WT	Total: *n* = 6/group	FGFR antagonists restored defective mandibular bone repair in a mouse model of osteochondrodysplasia	Morice et al. 2025 [65]
	Mouse	Femur	PFA, MS	Paraffin	Visium CytAssist Visium HD	Femur defect, postop day0 vs. day5 vs. d15	Total: *n* = 2/timepoint ST: *n* = 2/timepoint	By combining scRNAseq and ST, the activation and differentiation of periosteum progenitor cells was characterized	Wang et al. 2025 [63]

Abbreviations: *DV200* Distribution Value 200, *EDTA* Ethylenediaminetetraacetic acid, *LivE* Local in vivo Environment, *MS* Morse’s solution, OA osteoarthritis, OAA oxaloacetate, *PFA* Paraformaldehyde, *RIN* RNA integrity number, *PC* post conception week

Within this review we provide an overview of recent ST applications and advances in bone research. The included studies cover three main topics: skeletal development and maintenance, bone-related disorders, as well as fracture healing. The review is structured into three sections covering (1) applications of ST to bone, (2) the ST platforms being used and (3) considerations for the generation and analysis of ST data. We provide an overview on ST workflows (from sample preparation to data analysis) and discuss recent advancements, current challenges and future directions of ST applications. By highlighting integration of further methods to ST workflows for bone, our perspective outlook supports emerging multi-omics and multi-modal imaging approaches to advance translational-targeted ST applications in the fields of orthopaedics and musculoskeletal research.

## Applications and Advances of ST in Bone

In recent years, the first ST applications have been conducted on skeletal samples obtained from preclinical and clinical studies (Table 1). The studies have employed ST to capture and advance our understanding of bone development and maintenance, bone-related disorders as well as fracture healing. These processes are tightly regulated involving diverse cell types, intracellular signalling pathways as well as the local microenvironment. Bulk and scRNA-seq have shed light on the cellular aspects of these processes [3, 38, 39]; however, only with the advent of ST has it become possible to capture the transcriptome within the intact local microenvironment [40, 41].

### ST-based Advances in Bone Development and Maintenance

In a first effort to apply ST to bone, Baccin et al. employed a laser-capture-microdissection (LCM)- based approach to study the molecular, cellular and spatial bone marrow niche organization on cryo-sections from mouse femurs [42]. Specifically, they succeeded in isolating bone marrow (BM) pieces, with an average area of 14’500 µm^2^ (corresponding to around 200–300 cells), in different BM districts and applied them to RNA-seq. By integrating scRNA-seq data from the same mouse cohort, Baccin et al. revealed the molecular, cellular and spatial organization of BM niches [42]. Their data demonstrated that Cxcl12-abundant-reticular (CAR) cell subsets (Adipo-CAR and Osteo-CAR) differentially localize to sinusoidal and arteriolar surfaces, acting locally as ‘professional cytokine-secreting cells’ and thereby establishing peri-vascular micro-niches [42]. With the development of commercial platforms, further studies have applied ST, advancing the molecular understanding of cellular heterogeneity within BM niches [43, 44]. Xiao et al. found that different types of skeletal stem and progenitor cells reside in specific locations of the bone [44]. By combining ST data with bone marrow scRNA-seq and predictive modelling packages to deconvolve the larger spatial spots into their cellular constituents, they were able to spatially localize skeletal stem and progenitor cells (SSPC) subtype populations. Using correlative analyses, Xiao et al. mapped out cellular subtypes enriched within these SSPC-containing spatial spots and showed that Pdgfra^+^Sca1^+^ SSPCs preferentially localize to the periosteal surface; Cxcl12^+^Lepr^+^ SSPCs, on the other hand, were enriched within the marrow. Using cell‒cell interaction analyses, spatial gene expression, and spatial-time analyses, they showed that signals from blood vessels and bone surfaces create local microenvironments in the marrow by controlling metabolism and key growth pathways [44]. Furthermore, ST enabled the identification of specific molecular features during bone development using mouse and human samples [45–48]. By combining ST with bulk, scRNA-seq and transposase-accessible chromatin sequencing, Pina et al. identified distinct spatiotemporal expression patterns of key osteogenic marker genes indicative of different palate developmental stages [47]. Specifically, they observed a transcriptional shift between mouse embryonic days 14.5 and 15.5 which marks the onset of osteogenesis and indicates different developmental stages [47]. An important finding of their comprehensive transcriptomic profiling analysis was the identification of up-regulated marker genes (*Deup1*,* Dynlrb2*,* Lrrc23*) not previously reported in the process of palatal fusion.

Recently, comprehensive efforts in combining sequencing, imaging and computational tools have advanced the establishment of atlases for human embryonic development of skeletal tissues [49–51]. For example, Zhang et al. applied scRNA-seq and ST to detail human embryonic limb development across space and time. Subsequent comparison with scRNA-seq from mouse embryonic limbs indicated substantial cross-species conservation [49]. Recently, the data from Zhang et al. has been integrated in a multi-omics atlas of human embryonic skeletal development by the same group [50].

In an effort to characterize refined in vitro systems, Gribaudo et al. showed the recapitulation of spinal cord/spine co-morphogenesis by employing ST and scRNA-seq in self-organizing models of human trunk organogenesis [52]. This work highlights the potential of ST to support and drive advances towards integrated complex in vitro models of bone development and maintenance.

### ST-based Advances in Bone-Related Disorders

ST applied to human biopsies have characterized distinct localized morphological and transcriptomic changes in bone-related disorders such as osteoarthritis and different types of bone tumours and metastasis ([53–56]; see Table 1). ST has shown to be particularly suited for addressing the spatial heterogeneity of bone tumour microenvironments (TME) with dynamically changing and interacting cell types within the extracellular matrix [11, 37, 57]. ST has identified distinct TMEs of lytic and blastic bone metastasis in prostate cancer patients as well as distinct cancer associated fibroblast (CAF) subpopulations in the TME of chordoma patients enabling for subsequent multi-omics follow-up [53, 54, 56]. Associated with the fast progression in the field, several preprints further support und underscore the importance of integrating ST-based workflows to advance our molecular understanding of bone-related disorders. For example, Sudupe et al. spatially profiled the bone marrow from a multiple myeloma mouse model and uncovered heterogeneous malignant plasma cell niches and associated inflammatory signalling gradients [58, 59]. These gradients correlated with immune cell exhaustion and disease severity, pointing to new therapeutic targets within the TME [58]. From a clinical perspective, integration of ST to diagnostic workflows could facilitate deep spatial phenotyping of biopsies from patients with bone-related disorders and enable targeted treatments [60].

### ST in Fracture Healing

Fracture healing is a spatio-temporally regulated process involving multi-tissue crosstalk during the different healing phases [14]. Recent sample pre-treatment protocols extend the application range of ST from lowly mineralized samples during digit regeneration [61] to mineralized callus in fracture healing studies via optimization of fixation and decalcification [14, 16, 35]. By applying ST and scRNA-seq at two different healing-phase specific timepoints (d5, d15) in a mouse fracture model, Wang et al. characterized the activation and differentiation of periosteum progenitor cells and explored potential receptor‒ligand pathways that mediate cellular interactions using CellChat [62, 63]. Particularly high levels of cellular communication activity were detected in areas of regenerative mesenchymal progenitor cells (rMPCs) and macrophages as fracture healing progressed, highlighting an active intercellular dialog critical for the healing process [63].

ST is also increasingly applied to spatially assess molecular features associated with impaired healing conditions. A preliminary report by Jiang et al. indicated that impaired fracture healing in metastatic breast cancer is associated with downregulation of matrix and structural regulators in callus tissue one week after fracture [64]. Morice et al. used ST to characterize mandibular defect healing in a mouse model of hyperchondrodysplasia (Fgfr3^N534K/+^) harbouring a gain of function mutation in fibroblast growth factor receptor 3 (FGFR3) [65]. They showed reduced levels of osteogenic markers in the callus and revealed key molecular disruptions underlying the observed impaired bone repair such as autophagy, apoptosis and mitogen-activated protein kinase pathway*s*. Interestingly, FGFR3 antagonists were able to restore the defective mandibular bone repair indicating a potential treatment option for patients with FGFR3-osteochondrodysplasia. Similarly, Rois et al. applied ST to investigate impaired fracture healing in the *Postn*-cre;*Nf1*^fl/–^ (*Nf1*^Postn^) mouse model of pseudoarthrosis providing spatially-resolved molecular evidence of impaired BMP signalling, thereby supporting potential off-label BMP2 use as a therapeutic intervention [66]. Collectively, these studies highlight the potential of ST integration to preclinical fracture healing studies to drive translational-targeted treatments for impaired healing conditions.

ST is also of interest for the analyses of local responses to biomaterials during bone regeneration. A recent study by Wan et al. demonstrated how a subset of mesenchymal stem cells (MSCs) can orchestrate the osteoimmune microenvironment during collagen/nanohydroxyapatite-mediated bone regeneration [67].

In an effort to link mechanical and molecular features during fracture healing, Mathavan et al. developed a ST-based mechanomics platform integrating time-lapsed in vivo micro–computed tomography [68], spatial transcriptomics [14] and micro–finite element analysis to a femur defect loading model in mice [69, 70]. It allowed to spatially profile the effect of mechanical loading on the transcriptome in high/low strained callus regions during the remodelling phase of in fracture healing [70]. This mechanomics platform can serve as an example and provides a basis for advancements in multi-modal imaging approaches.

Currently, a comprehensive spatial molecular atlas covering the different bone compartments across developmental stages and maintenance, bone-related disorders and fracture healing is still an unmet need. Isolated studies and tissue-specific atlases have offered valuable insights into specific bone compartments or disease states. However, systematic and integrative spatial transcriptomic efforts are essential for such atlases to serve as foundational references to guide future bone research and therapeutic development.

## Considerations for the Generation and Analysis of ST Data in Bone

**Fig. 1:**
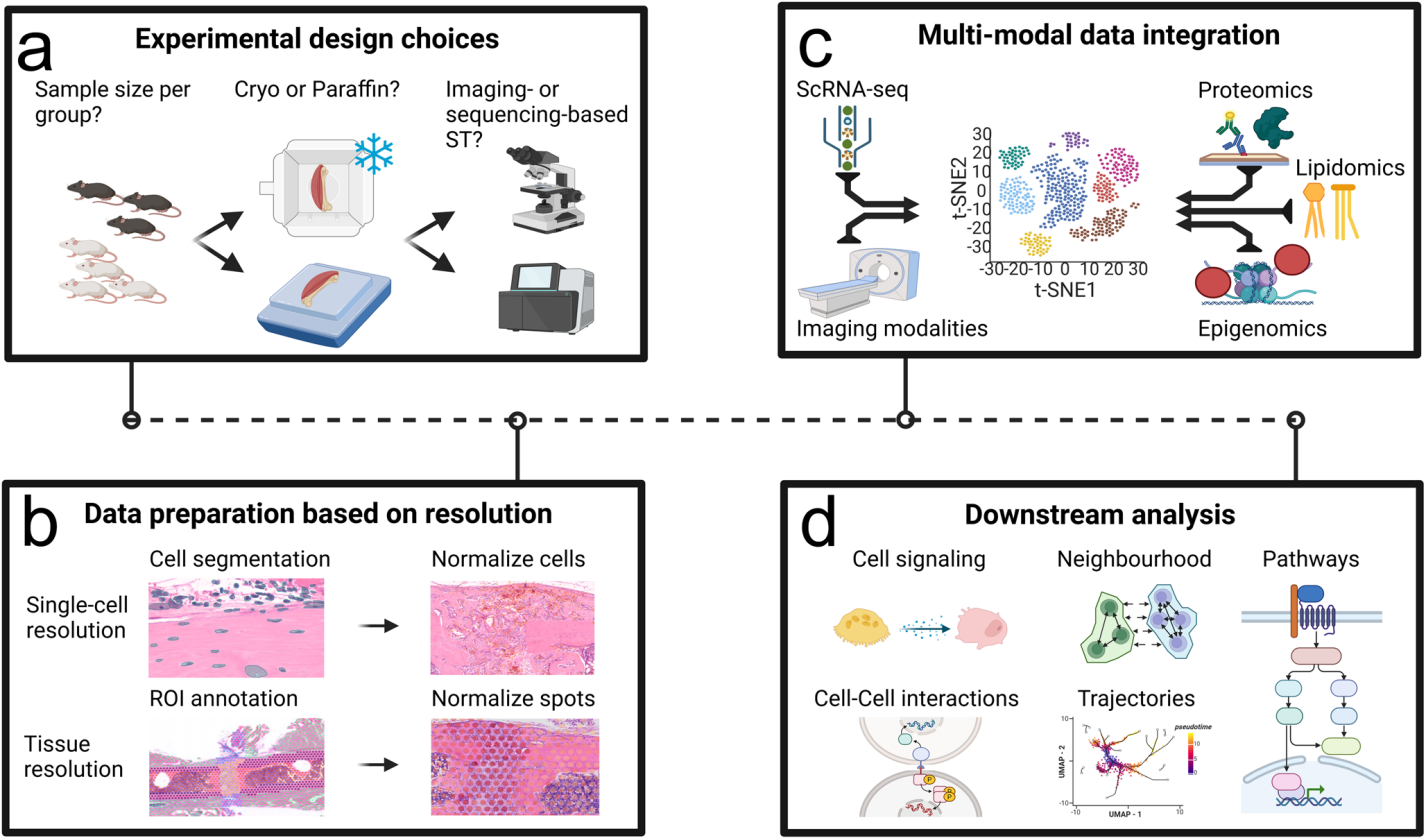
Consideration for ST in Bone. (**a**) Experimental design choices consisting of type and number of samples per group (left), embedding strategies including cryo- or paraffin-embedding (middle) and the choice of imaging-based vs. sequencing-based ST platforms (right). (**b**) Resolution-dependent data preparation. For single-cell resolution, cell segmentation can be used and single cells normalized (top row). Regions of interest (ROIs) can be annotated in ST data with tissue-resolution and individual spots are normalized (bottom row). (**c**) Imaging and omics modalities, including single-cell RNA sequencing (scRNA-seq), proteomics, lipidomics and epigenomics can be integrated and visualized in a t-distributed Stochastic Neighbour Embedding (t-SNE). (**d**) Downstream analysis includes a combination of cell signalling, cell-cell interaction, neighbourhood, trajectory and pathway analysis. Created in https://BioRender.com

Studies applying ST in bone have employed a variety of set-ups and combinations related to technology platforms, sample preparation protocols and data analyses workflows (Table 1). We provide an overview of key features of the ST platforms used in bone (Table 2), applicable sample preparation protocols and data analyses procedures, highlighting their current status as well as ongoing and future developments (Fig. 1).

**Table 2:** ST platforms applied in bone: Categories, features and requirements

	Platform	Backbone technology	Resolution	Whole transcriptome coverage	Applicable sample types	Assay input	Recommended sample QC	Company	Pros	Cons	Technology reference
Imaging-based	**CARTANA HS**	ISH	Single cell	No (up to 600 transcripts)	FF/FFPE	Section on histology slide	DAPI	-	Single cell resolution	Requires prior knowledge of genes of interest	Ke et al. 2013 [72]
	**Xenium**	ISS, ISH	Single cell, Subcellular (200 nm)	No (up To 5000 transcripts)	FF/FFPE	Section on Xenium slide	DAPI and H&E, DV200 > 30%	10x Genomics	Subcellular resolution	Requires prior knowledge of genes of interest	Janesick et al. 2023 [71]
Combined	**GeoMx Digital Spatial Profiler**	ISH, ST	Region specific	Yes	FF/FFPE	Section on positively charged histology slides	RNAscope	Nanostring	Multiple ROIs within same tissue section can be taken; Simultaneous protein detection	Fluorescence signals pooled in ROI	Merritt et al. 2020 [78]
Sequencing-based	**Tomo-seq**	RNA-seq of individual histology sections	Section-specific	Yes	FF	1 histology section collected in tube	NA	NA	High read depth, 3D reconstruction	Low spatial resolution	Junker et al. 2014 [73]
	**LCM-seq**	LCM, RNA-seq of individual ROIs	Region-specific	Yes	FF/FxF/FFPE	ROIs isolated by LCM collected in tube	NA	NA	High read depth	Physical dissection of ROI	Nichterwitz et al. 2016 [74]
	**Visium v1**	ST: 3’ gene expression-based approach, mRNA released from tissue section directly binds to the poly(dT) region of adjacent capture probes on the array	~ 55 μm spot diameter	Yes	FF	Sections on Visium slide	FF: RIN ≥ 7	10x Genomics	Applicable to diverse species	Deconvolution needed for single-cell information	Stahl et al. 2016 [77]
	**Visium v2**	ST: probe-based approach (CytAssist), ~ 1 pair (mouse) or ~ 3 pairs (human) of specific probes per targeted gene	~ 55 μm spot diameter	Yes	FF/FxF/FFPE	Sections on Visium slide or sections on histology slide (CyAssist)	FF: RIN ≥ 7, ≥ 4 (CytAssist) FxF: DV200 ≥ 50% FFPE: DV200 ≥ 50%, ≥ 30% (CytAssist)	10x Genomics	Compatibility with CytAssist, therefore no direct section placement on spatial slide required, enables preselection of sections on histology slides	Deconvolution needed for single-cell information	Stahl et al. 2016 [77]
	**Visium HD WT**	ST: Probe-based approach, ~ 3 pairs of specific probes per targeted gene	2 × 2 μm grid	Yes	FF/FxF/FFPE	Sections on histology slide (CyAssist)	FF: RIN ≥ 4 FxF: DV200 ≥ 50% FFPE: DV200 ≥ 30%	10x Genomics	Lossless grid instead of interspaced spots, single cell resolution	Higher costs compared to Visium v1/v2	Yin et al. 2024 [76]

Abbreviations: *DAPI* Nuclear stain 4’,6-diamidino-2-phenylindole, *DV200* Distribution Value 200, *FF* Fresh Frozen, *FxF* Fixed Frozen, *FFPE* Formalin-fixed paraffin-embedded, *H&E* Hematoxylin and eosin stain, *ISH* In situ hybridization, *ISS* In situ sequencing, *LCM* Laser Capture Microdissection, *ROI* Region Of Interest, *RIN* RNA integrity number, *Tomo* Tomography

### ST Platforms Applied in Bone

Imaging and sequencing-based platforms have been used for ST in bone (Tables 1 and 2). Imaging-based technologies employ in situ hybridization as their backbone technology, enabling the simultaneous transcript detection of hundreds to thousands of targets [10, 13, 71, 72]. First sequencing-based approaches applied RNA-seq to single histology sections (Tomo-seq [73]) or to regions of interest (ROIs) on tissues captured by LCM (LCM-seq [74]). Recent sequencing-based ST platforms combine spatially barcoded arrays with next-generation sequencing (NGS) to determine the locations and expression levels of RNA transcripts in tissue sections. The available ST platforms differ in array design, which largely determines their spatial resolution [75–77]. Combinatorial ST platforms integrate sequencing- and imaging-based approaches, e.g. via combining barcoded probes and ROI selection to determine spatial transcript location [78]. Table 2 provides an overview of the specific features of the imaging and sequencing-based ST platforms, that have been applied to bone. The platforms differ in the backbone technology (e.g. 3’ gene expression vs probe-based setups), resolution (subcellular - whole histology section), transcriptome coverage (several hundred - whole transcriptome), applicable sample types (fresh frozen - FF, fixed frozen - FxF, FFPE), assay input (section on histology slide vs specific ST slide), and required sample specifications and quality (e.g. RNA integrity number - RIN, Distribution Value 200 - DV200; Table 2). For a comprehensive general review on imaging-and sequencing-based ST technologies see [75]. Current ST platform developments are focused on increased resolution and transcriptome coverage as well as their applicability to a wider range of species.

### Bone Sample Preparation for ST

The development of sample preparation protocols has been key for successful application of ST to mineralized musculoskeletal samples [14, 16, 35]. By optimizing the workflows and settings (e.g. reagents, incubation times, temperature) for each step (sample preparation, embedding, sectioning, imaging) the protocols are targeted towards maintaining sample RNA quality enabling the selection of the most promising sections for ST. General recommendations include an RNase-free working environment, the use of molecular grade and RNase-free reagents as well as the use of RNase-free or MilliQ water. Common critical parameters for tissue harvesting have been described, such as the time from euthanasia to sample placement in the respective solution, the pre-cooling of solutions and performing the sample extraction on ice. Bone samples applied to ST have been either embedded in cryo-medium or paraffin (Table 1).

Fresh-frozen (FF) approaches with direct *cryo-embedding* of samples seem particularly suited for non- and lowly mineralized tissue such as chordoma [54, 56] or samples obtained during early developmental stages [49–51, 61] and regeneration [67]. Baccin et al. provided a detailed PFA-based protocol for FxF cryo-samples (30 min ice-cold PFA, 2 h 15 + 30% RNase-free sucrose solution, sample freezing in methylenbutane/dry ice bath) [42]; however, subsequent studies favoured FF approaches for ST application in bone. To keep tissue morphology during sectioning, specific recommendations for cryotome settings have been provided for bone samples (e.g. blades, temperature [42]). Recommended sample quality checks include the assessment of the RIN, which was shown to provide a good approximation for subsequent successful ST application (for specific thresholds and platform requirements see Table 2). Additionally, nuclear stains (e.g. DAPI) in combination with H&E have been used to assess nuclei quality, tissue necrosis and morphology, as predictors of ST success. Imaging and sample storage parameters (temperature, duration) were characterized as further factors impacting ST in bone. To enable ST application of already embedded and partially degraded bone samples, Mirzazadeh et al. recently presented an RNA-Rescue Spatial Transcriptomics (RRST) protocol [79]. Their procedure includes a fixation step, which makes the samples compatible with ST FFPE workflows not requiring high RNA quality/RIN values [79]. Future ST developments for cryo-embedded bone samples could include Kawamoto film-based cutting approaches, which have been successfully used for spatial lipidomics of spine samples [80]. We see the potential of such methods to enable the application of ST to FF sections without the need of decalcification, thereby circumventing the risk of associated RNA degradation.

Bone *paraffin-embedding* protocols for ST application commonly include tissue fixation and decalcification steps [14, 16]. Fixation only protocols (PFA, formalin) have been used for non- and lowly mineralized tissue such as bone tumours [53] or samples obtained during early developmental stages [47]. With recent optimizations of decalcification procedures, ST application to fully mineralized samples have been achieved [36, 37, 39]. Studies have successfully employed different decalcification agents (ethylenediaminetetraacetic acid - EDTA, Morse’s solution, formic acid, Table 1) prior to ST. Fixation and decalcification parameters (reagent, temperature, duration) can be seen as essential factors for successful ST in bone [15, 81, 82]. ST studies in bone have employed the following reagent-specific fixation parameter ranges for formalin (10%, 4 °C, 16–48 h, +/-shaking) and PFA (4%, 4 °C or RT, 30 min–24 h, +/-shaking). Applied reagent-specific decalcification parameters have covered the following ranges for EDTA (10–20%, 4 °C or RT, 3days-3weeks), Morse’s solution (20 h, RT) and formic acid (4 °C, 16 h). This indicates that a wide range of bone pre-treatment protocols can be compatible with ST. A good proxy for RNA quality of FFPE samples used for ST is the DV200 (percentage of RNA sequences > 200 nucleotides) [14, 15]. Additionally, studies have indicated further crucial factors and optimization points, such as the use of buffered solutions for sample pre-treatment steps, and controlled conditions during sample storage (4 °C, low humidity). This is in line with our own observations that RNA quality (DV200) and sequencing depth seem to be associated with treatment and storage conditions. Another factor determining ST success, is tissue attachment on the histology slide. In particular, the high temperature during decrosslinking steps (which makes RNA accessible in fixed tissues) poses a risk for tissue detachment during sample preparation. Two approaches have been followed to preserve tissue integrity: (1) Mirzazadeh et al. included an additional baking step [79] and based on our own experience, (2) the usage of hydrogel-coated histology slides can be recommended to maintain section morphology.

Comparative studies have characterized similarities and differences of samples applied to cryo- and FFPE based methods in down-stream analyses [79]. Future benchmark studies could provide additional means to advance ST in bone, align preparation protocols, and improve comparability between studies.

While a range of treatment protocols for bone samples have been previously published, common strategies focus on restricting the fixation and decalcification times at controlled temperature to maintain RNA quality; approximately 1 day of fixation and 10 days of decalcification (both at 4 °C) seem to represent a rough consensus when it comes to processing mouse bones for ST. These treatment periods are supported by our own experience.

### Data Analysis of ST in Bone

Methods to analyse ST data derived from bone tissue have been adapted from bulk and scRNA-seq workflows. Bulk RNA-seq identifies differentially expressed genes across entire tissues or conditions. The amount of RNA captured by bulk RNA-seq allows for expression quantitative trait loci (eQTL) analysis as well as measurement of allele-specific expression [19]. ScRNA-seq reveals cell subpopulations and their interactions, while ST adds spatial context to show where these cells reside in the tissue. The optimal unit for ST consists of a cell and its local microenvironment [23]. The spatial units are based either on pixels (imaging-based ST) or predefined regions part of the ST workflow (sequencing-based ST, e.g. spots in Visium). Independent of the ST technologies, a gene-count matrix links spatial units with their transcriptome [83]. For this type of data, multiple computational frameworks are available [84–86]. Multi-sample comparisons are challenged by small sample sizes so far used in ST-based bone studies (Table 1). To reliably detect differentially expressed genes between two conditions, Shui et al. developed PoweREST for power estimation of Visium data [87]. Such tools are a first step to address sample size calculation as part of the experimental design. For integration of ST across samples and tissues we refer to Guo et al. [24].

In the following, we summarize the current state of *data preparation*, *data integration* of different omics approaches, and *downstream analysis* methods.

Within the context of *data preparation*, annotations facilitate the generation of biologically meaningful spatial units. Subsequent filtering purges the data from bad-quality spatial units and robust normalization ensures that the observed differences in gene expression reflect biological variations rather than technical artifacts. *Annotation*s depend on the resolution of the ST data (Fig. 1b) and lay the groundwork for reliable analyses. Most sequencing-based technologies lack single-cell resolution (Table 2). Therefore, ROIs are annotated manually and subsequently used for differential gene expression. With high-resolution ST (e.g. Visium HD), cell annotations can be achieved on sequencing-based ST by binning to cells [88]. Imaging-based ST requires advanced segmentation strategies which often rely on a nucleus or a membrane stain [89, 90]. For Xenium data, a benchmark study by Salas et al. proposes a combination of Baysor and Cellpose [89–91]. To et al. exemplify cell segmentation in bone using Cellpose [50, 91]. Recently, segmentation-free approaches are also being developed to attribute transcripts to cells [92]. These are based on the transcript fluorescence signal to define the cell shape which allows for cross-platform generality, scalability and precision [92]. In simpler terms, annotations depend on the resolution of the ST technology applied (see Table 2). For single-cell resolution technologies, transcripts can be assigned to single cells by cell segmentation. For tissue resolution technologies, ROIs can be annotated based on location, tissue morphology or local gene expression patterning (Fig. 1b). Annotations are especially important in multi-tissue musculoskeletal samples to account for tissue-specific RNA quality. *Filtering* can be achieved by scoring each spatial unit based on mitochondrial gene percentage, gene counts, and total RNA counts [93]. Recently, Totty et al. presented a spatially aware quality control (QC) using a k-nearest-neighbour approach [94]. *Normalization* algorithms developed for scRNA-seq data should be applied cautiously to ST, as library size is often reflected by the underlying tissue structure [95]. Normalizing for library size may therefore obscure real spatial insights with impact on biological interpretation of downstream analysis [24, 30]. It is recommended to use cell volumes (or cell areas as a proxy) to normalize after segmentation [30]. For cell-annotation-free data, spatially aware normalization approaches exist [25]. Currently, there are various preprints available on topics such as ST quality controls, segmentation and normalization approaches, highlighting the need for future benchmarks that include ST data from bone. Filtering and normalization steps are essential quality checks and adjustments that help ensure ST output truly reflects biological differences, not just technical noise. This is especially important in bone, which is composed of multiple tissues with varying cell densities and cell sizes.

*Data integration* stands in contrast to bulk sequencing and scRNA-seq as ST by itself combines two data modalities: (1) Transcript counts based on sequencing reads or fluorescence signals and (2) morphology based on tissue structure including neighbourhood metrices. However, ST lacks information about cell subpopulations, epigenetic state of a cell, protein and lipid abundance. This can be provided by integrating scRNA-seq, ChIP-seq, spatial proteomics, and spatial lipidomics (Fig. 1c) [45]. Integration of additional imaging modalities, such as micro-CT, have been demonstrated by Mathavan et al., who superimposed mechanical strain from micro-CT–derived finite element models onto ST data [70]. Similarly, it would be possible to use 2-photon-microscopy imaging of inorganic molecules and pH [96]. Due to a high read depth, scRNA-seq data sets are used to deconvolute the mixed signals in sequencing-based ST, allowing researchers to infer single-cell expression profiles in ST data. Benchmark studies have been conducted to assess the performance of deconvolution algorithms [32, 97]. There are also reference-free methods like STdeconvolve which provide an alternative to circumvent the bias towards the scRNA-seq data input [98]. This could be a viable option in tissues such as bone where only few data sets are available. For a comprehensive review of deconvolution methods see Gaspard-Boulinc et al. [21]. Integration of multi-omics data sets from different layers, incl. epigenetics, RNA, protein and lipids, requires substantial bioinformatic and computational expertise [99]. Various strategies exist from clustering based on spatially variable genes to network-based and deep learning approaches [100]. To dive deeper into the topic, we recommend the review by Acharya & Mukhopadhyay [100]. In other words, data integration involves linking information from multiple sources such as transcript, protein or lipid abundance, tissue structure, or even mechanical forces (Fig. 1c). Integrating omics data in this manner can provide a comprehensive understanding of the molecular mechanisms guiding bone tissue in both health and disease.

Given the breadth of *downstream analysis* approaches, we refer to several reviews which include methods to investigate local neighbourhoods, cell signalling and cell-cell interactions, pathway analysis and trajectory analysis (Fig. 1d) [6, 18, 20, 22, 23, 26, 28]. The following examples are exclusive to bone. Xiao et al. studied cell signalling and cell-cell interactions by examining metabolic and morphogenetic signalling gradients across multiple tissues to find bone marrow niches [44]. Pathway analysis by Rios et al. indicated distinct regulation of intercellular signalling at the fibrocartilaginous-osseus junction in fracture-pseudoarthrosis [66]. Spatiotemporal trajectory analysis has also been implemented in bone ST data [44, 46]. While these downstream analyses rely on scRNA-seq data, ST in bone has effectively demonstrated the allowance for complex local niche analysis (Table 1).

While downstream analysis strategies are expected to rapidly advance, we would like to emphasize the importance of validating ST results. Although concordant spatial patterns of RNA and protein strengthen biological conclusions, protein abundance does not directly correlate with mRNA levels due to post-transcriptional regulation [18]. In contrast, RNA in situ hybridization (RNA ISH) methods, such as RNAscope, are well-suited for ST data validation, as they allow direct visualization of specific RNA transcripts at single-molecule resolution within intact bone tissue [47, 52]. Ideally, such ISH-based approaches are applied to sections directly adjacent to the ST section providing options for co-localization analyses. To sum up, downstream analysis uses spatial data to explore cell-cell interactions and cell signalling, pathway activity, as well as cell trajectories (Fig. 1d). Methods for such analyses are under development, and it is important to confirm and validate findings from ST using complementary RNA detection methods such as RNA ISH.

## Conclusion and Perspective Outlook

ST plays a leading role in advancing bone research. Recent ST platforms and the development of sample preparation protocols applicable to mineralized tissue have been driving ST application in preclinical and clinical bone studies. ST has enabled for spatiotemporal analyses in intact bone and fracture healing with the development of tissue specific skeletal ‘atlases’.

A limitation of the currently available ST data sets in bone is the low sample size used in the studies. Another aspect to consider is the lack of standardized data analyses pipelines for ST in bone, making comparisons between data sets difficult. We therefore stress the importance of pre-experimental design choices (Fig. 1a) with appropriate experimental groups, sample size calculations, selection of ST platforms as well as sample preparation and imaging protocols. While these are the building blocks for the data acquisition, subsequent image annotations and normalization approaches (Fig. 1b) should be carefully considered, depending on the platform of choice, based on tissue or cell resolution. ST requires spatially resolved algorithms for normalization and downstream analyses, especially in complex tissues such as bone.

Eventually, the goal of ST analysis is to leverage the spatial information for niche analysis within the local cellular microenvironment (Fig. 1d), which often relies on multi-omics approaches to enrich the ST data set (Fig. 1c). These can range from bulk and single-cell transcriptomic data to epigenetic, proteomic or lipidomic data. Furthermore, we see a potential of co-registering additional imaging modalities for multi-modal ST approaches in bone. While data interpretation must be taken with caution, we consider ST a cornerstone technology for unravelling bone’s spatial and molecular complexity. ST has started to accelerate discoveries in bone development, bone-related disorders, and fracture healing. Via integration into emerging multi-modal and multi-omics approaches, ST has the potential to advance translational-targeted applications in the fields of orthopaedics and musculoskeletal research.

## Key References

- Wang S (2025). Resolving the bone - optimizing decalcification in spatial transcriptomics and molecular pathology. J Histotechnol 48(1):68–77.
  - ○ This review provides an overview of sample preparation protocols for spatial transcriptomics in bone.
- Gulati GS, D’Silva JP, Liu Y, Wang L, Newman AM. Profiling cell identity and tissue architecture with single-cell and spatial transcriptomics. Nature Reviews Molecular Cell Biology. 2025;26(1):11–31.
  - ○ This review provides an overview of spatial transcriptomics data analyses methods to profile tissue architecture.
- Zhang B, He P, Lawrence JEG, Wang S, Tuck E, Williams BA, Roberts K, Kleshchevnikov V, Mamanova L, Bolt L, Polanski K, Li T, Elmentaite R, Fasouli ES, Prete M, He X, Yayon N, Fu Y, Yang H, Liang C, Zhang H, Blain R, Chedotal A, FitzPatrick DR, Firth H, Dean A, Bayraktar OA, Marioni JC, Barker RA, Storer MA, Wold BJ, Zhang H, Teichmann SA (2024). A human embryonic limb cell atlas resolved in space and time. Nature 635:668–678.
  - ○ This paper provides an example for the development of a spatial transcriptomics-based tissue-specific cell atlas. By combining scRNA-seq and ST limb development, cell diversification & cross-species conservation were demonstrated.
- To K, Fei L, Pett JP, Roberts K, Blain R, Polański K, et al. (2024). A multi-omic atlas of human embryonic skeletal development. Nature. 635(8039):657-67.
  - ○ This paper provides an example for the development of a multi-omic atlas. Via combining sequencing and imaging-based technologies a multi-omic atlas of embryonic development was created.
- Mathavan N, Singh A, Marques FC, Günther D, Kuhn GA, Wehrle E, et al. (2025). Spatial transcriptomics in bone mechanomics: Exploring the mechanoregulation of fracture healing in the era of spatial omics. Sci Adv 11(1):eadp8496.
  - ○ This paper provides an example for a spatial transcriptomics-based multi-modal study approach. In an effort to link mechanical and molecular features during fracture healing, a spatial transcriptomics-based mechanomics platform was developed integrating time-lapsed in vivo micro–computed tomography, spatial transcriptomics and micro–finite element analysis to our femur defect loading model in mice. Via linking spatial transcriptomics to the local mechanical microenvironment, spatial profiles in high/low strained callus regions were characterized.

## Data Availability

No datasets were generated or analysed during the current study.

## Declarations

All reported studies/experiments with human or animal subjects performed by the authors have been previously published and complied with all applicable ethical standards (including the Helsinki declaration and its amendments, institutional/national research committee standards, and international/national/institutional guidelines).
